# Magnetic Nanoparticles in Biology and Medicine: Past, Present, and Future Trends

**DOI:** 10.3390/pharmaceutics13070943

**Published:** 2021-06-24

**Authors:** Deanna D. Stueber, Jake Villanova, Itzel Aponte, Zhen Xiao, Vicki L. Colvin

**Affiliations:** 1 Center for Biomedical Engineering, School of Engineering, Brown University, 171 Meeting Street, Providence, RI 02912, USA; deanna_stueber@brown.edu (D.D.S.); jake_villanova@brown.edu (J.V.); itzel_aponte@alumni.brown.edu (I.A.); 2 Department of Chemistry, Brown University, 324 Brook Street, Providence, RI 02912, USA; zhen_xiao@brown.edu

**Keywords:** magnetic nanoparticles, iron oxide, magnetic resonance imaging, magnetothermal heating, magnetic separation, superparamagnetic, drug delivery, gene delivery, magnetic particle imaging, microfluidics

## Abstract

The use of magnetism in medicine has changed dramatically since its first application by the ancient Greeks in 624 BC. Now, by leveraging magnetic nanoparticles, investigators have developed a range of modern applications that use external magnetic fields to manipulate biological systems. Drug delivery systems that incorporate these particles can target therapeutics to specific tissues without the need for biological or chemical cues. Once precisely located within an organism, magnetic nanoparticles can be heated by oscillating magnetic fields, which results in localized inductive heating that can be used for thermal ablation or more subtle cellular manipulation. Biological imaging can also be improved using magnetic nanoparticles as contrast agents; several types of iron oxide nanoparticles are US Food and Drug Administration (FDA)-approved for use in magnetic resonance imaging (MRI) as contrast agents that can improve image resolution and information content. New imaging modalities, such as magnetic particle imaging (MPI), directly detect magnetic nanoparticles within organisms, allowing for background-free imaging of magnetic particle transport and collection. “Lab-on-a-chip” technology benefits from the increased control that magnetic nanoparticles provide over separation, leading to improved cellular separation. Magnetic separation is also becoming important in next-generation immunoassays, in which particles are used to both increase sensitivity and enable multiple analyte detection. More recently, the ability to manipulate material motion with external fields has been applied in magnetically actuated soft robotics that are designed for biomedical interventions. In this review article, the origins of these various areas are introduced, followed by a discussion of current clinical applications, as well as emerging trends in the study and application of these materials.

## 1. Introduction

Magnetism has been linked to medicine for thousands of years. It is thought that the Greek scientist and astronomer, Thales of Miletus, was the first person to apply magnetic materials to organisms as early as 624–547 BC. His work led to a cultural belief in the healing powers of lodestones that persisted for centuries [1]. In the 14th century, the Swiss doctor and alchemist, Paracelsus, wrote the *Volumen Medicinae Paramirum* which detailed how to manipulate the health of a body using magnets. After seeing the way that magnets could attract iron, he hypothesized that magnets could be used to attract diseases from the body in the same way [1]. Several hundred years later, in 1892, the first definitive study of magnets on organisms was completed. Five humans and one dog were exposed to magnetic fields of roughly several thousand gauss or several thousand times the earth’s magnetic field, but no measurable effect was observed [2]. The first modern discussion of the prospects for magnetism in medicine was published in 1962 by Freeman et al., who predicted that magnetism would emerge as a powerful tool for biochemical analysis and medical diagnosis [3].

By the 1970s, the significance of magnetism in medicine was a reality in diagnostic imaging, but broader applications remained elusive until the development of nanotechnology. Magnetic resonance imaging (MRI) transitioned from the laboratory into the clinic in the early 1970s, and it was soon widely applied for detecting cancerous tumors [4]. Because of MRI scanners, doctors, for the first time, had access to instruments capable of applying large magnetic fields (B_o_ > 2 T) and this inspired many to explore how magnetism could be used for more than just imaging. Unfortunately, this avenue of research resulted in little new applications and conventional MRI imaging remained the dominant use of magnetism in medicine. However, with the advent of nanotechnology in the 1980s, native tissue could be transformed into magnetically responsive material using magnetic nanoparticles. This opened the door to a much wider set of potential medical applications. With appropriate surface functionality, magnetic nanoparticles, being typically less than a few hundred nanometers in dimension, could be used to label cells and biomolecules, thereby endowing tissues and other biological molecules with useful magnetic properties. The early applications of this new capability included the magnetic guidance of catheters for the treatment of bradycardic arrhythmia, movement of unerupted teeth in dentistry, and even magnetic intrauterine devices (IUD) for contraception [1].

**Figure 1:**
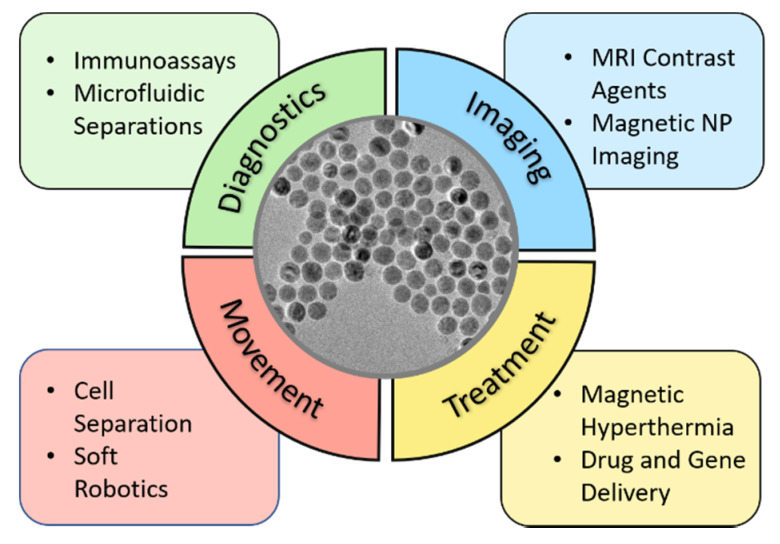
The applications of magnetic particles can be classified into four categories depending on the aim of the technology. Imaging and in vitro diagnostics are mature areas that have clinical relevance, while research into magnetic particles to treat disease or affect controlled motion of larger organelles, cells and biomaterials is at the pre-clinical stage. Abbreviations: Magnetic Resonance Imaging (MRI), Magnetic Nanoparticle (NP) Imaging Inset picture provided by Zhen Xiao, iron oxide (magnetite) nanocrystals d = 23 ± 2 nm.

Since the 1990s, there has been an explosion of research seeking to develop diverse medical applications for magnetic nanoparticles. In all cases, external magnetic fields interact with ferrimagnetic nanoparticles that can associate or interact with tissue, cells, or biomolecules allowing for applications from molecular imaging to magnetothermal heating (Figure 1). Superparamagnetic iron oxide nanocrystals (SPIONs) are central to these technologies; these materials (Figure 1) are made from iron oxide, but, because of their small dimensions, they do not exhibit any magnetization unless they are in an external magnetic field [5]. This is especially desirable for biological applications due to the decreased potential for aggregation in the absence of applied fields [6]. Figure 1 presents a loose classification of this large set of scientific literature based on the underlying goals of the technology: treatment, imaging, directed movement, and diagnostics. MRI imaging is a mature area of clinical practice, and the US Food and Drug Administration (FDA) has approved magnetic nanoparticles for use as MRI contrast agents, but most have been discontinued commercially [7]. Also notable is the widespread use of magnetic nanoparticles, typically referred to as “beads” by the analytical community, to facilitate immunoassays and other medical diagnostics. Emerging applications include cancer therapies, drug delivery, and magnetothermal schemes for disease therapy, as well as the controlled movement and direction of magnetic particles within organisms. While some of these examples have reached Phase 1 clinical trials, widespread clinical application has not yet been achieved [8,9,10].

To take full clinical advantage of these applications, it is vital to have practical systems for applying magnetic fields as well as highly responsive magnetic particles. Generating magnetic fields inside organisms that are large enough to affect particle movement is a challenge; particles move along the spatial gradient of a magnetic field and, often, field strengths are reduced to zero just a few millimeters away from a permanent magnet [11,12]. New designs for magnetic field application may make it possible to create larger field gradients that allow for the movement of materials far deeper in the body [13,14]. Additionally, clinical applications will demand models that can effectively predict magnetic particle movement in complex in vivo settings as such data are a necessary requisite for any clinical application. Finally, the clinical success of these new systems and models will require minimally toxic magnetic particles that are highly sensitive to even small external magnetic fields.

Here, four broad applications of magnetic nanoparticles in biology and medicine are surveyed: treatment, imaging, movement, and diagnostics (Figure 1). For treatment, magnetic nanoparticles are used to efficiently deliver various therapeutics, whether it is drugs, genes, or the particles themselves for magnetothermal heat treatment or as therapeutic catalysts. In clinical and preclinical imaging, magnetic nanoparticles are used as image-enhancing agents in MRI and magnetic particle imaging (MPI). Biomedically-relevant movement via the external field actuation of magnetic particles make the clinical translation of cell separation techniques and soft robotics more feasible. Finally, magnetic nanoparticles can be used to boost the diagnostic performance and throughput efficiencies of various immunoassays. Across these four broad fields, particular focus is given to iron oxide-based magnetic nanomaterials, because of their biocompatibility, versatility, and wide range of use. In each section, novel trends of magnetic nanoparticles are examined in light of their history and common uses within that field.

## 2. Treatment

### 2.1. Iron Oxide Catalyzed Cancer Therapies

Cancer treatment is one of the largest fields of biomedical research. Doxorubicin, gold, silver, and ferrite nanoparticles have all been studied for their cancer killing abilities, and they have been clinically applied to varying degrees. These therapies work through the increased generation and tuning of reactive oxygen species (ROS) in tumor regions that can induce apoptosis and cellular death [15]. Ferrite nanoparticles, specifically iron oxide nanoparticles, can be used for this purpose, due to their intrinsic peroxidase-like activity. By catalyzing the fenton reaction of H_2_O_2_, highly toxic hydroxyl groups, a type of ROS, are overproduced and cell death occurs. This was first discovered by Yan et al. in 2007 and, when combined with the magnetic targeting properties of these particles, it created considerable promise for the field [16]. Six years later, Zhang et al. took this knowledge and demonstrated the use of magnetic nanoparticles in tumor treatment [17]. Research has continued in this field focusing on the tunability of this characteristic through both manipulation of the particle itself and the external field acting upon it. While it is well studied that the catalytic activity can be tuned through particle size, composition, and morphology, recent trends in this field are focused on combining the biological and chemical properties through surface coatings and targeting molecules. For example, Thoidingjam et al. was able to synthesize phyllanthus emblica-coated iron oxide nanoparticles, which allowed for the stabilization of very small iron oxide nanoparticles (~6 nm), which are ideal for the overproduction of ROS in lung cancer cells [18]. Likewise, Pandey et al. synthesized poly-l-lysine-coated Fe_3_O_4_@FePt particles for the targeting of mitochondria through its pH responsiveness offering a targeted multimodal therapy for glioblastoma [19]. The next step for these treatments lies in optimizing their catalytic efficiency to increase the potential adoption into the clinical.

External electromagnetic fields, when absorbed by the ferrite material, can be used to boost the catalytic activity, thus increasing ROS production and decreasing the amount of ferrite material needed. Electromagnetic fields that are commonly studied for this purpose are alternating magnetic fields (AMFs) and X-ray [16]. AMFs were utilized by Wu et al., as they developed a magnetic hydrogel that is activated by a non-invasive external AMF to increase the production of ROS [20]. Similarly, Liu et al. synthesized novel graphene oxide- grafted iron oxide nanorings that have high magnetothermal properties. A significant increase in the ROS generation was observed when an AMF was applied [21]. The use of X-rays was studied when Klein et al. fabricated high stability, functionalized Coferrite and superparamagnetic magnetite particles that, when exposed to X-ray radiation, released either Fe^2+^ or Co^2+^ ions, leading to ROS production and cancer cell apoptosis [22]. As research continues in the area of tuning particle physical properties, external field manipulation advancements are a compounding asset in the fight against cancer.

### 2.2. Drug and Gene Delivery

Magnetic nanoparticles can be used to direct the delivery of drug and gene therapies in the body. A major challenge in pharmacology is the specific delivery of an agent to the disease site; most widely prescribed drugs that are taken orally or via intravenous injection are not targeted [23]. Consequently, it is estimated that less than 10% of the dose makes it to the organ of interest and even less to cellular targets [24]. The most common solution is to increase the delivered dose to assure sufficient drug concentration at the target site [3,25]. This inefficiency leads to off-target effects and toxicity, which can limit the clinical use of promising treatments. Additionally, non-selective delivery can also lead to negative immune responses at the site of administration.

Introducing selectivity into drug delivery is a general goal for all of pharmacology because of its broad relevance. One approach to increasing drug selectivity is by using nanoscale delivery systems, such as liposomes and polymeric nanoparticles, which possess cell-specific surface ligands. Several recent reviews have highlighted the common challenges that are faced by these non-magnetic biological and chemical targeting strategies [26,27,28,29,30,31,32,33,34,35,36]. Of these challenges, the most intractable is the body’s own physiological response to these foreign nanoscale systems, which quickly removes, metabolizes, and/or excretes them. Even with stealthy surface coatings that have only minimal protein interactions, nanoscale particles are still recognized and eliminated by the innate immune system [37]. As such, even with the most efficient targeted nanoscale delivery systems, only 2% of the drug payload is released at the target site [24].

**Figure 2:**
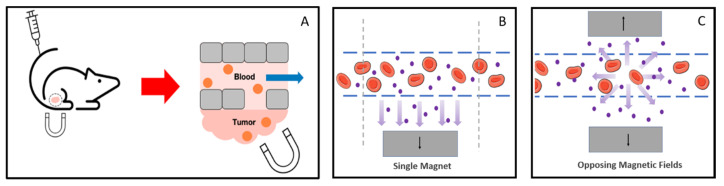
Schematic of magnetic drug delivery. (**A**) After a tail vein injection of magnetic nanoparticles, the materials collect at a site with a large external field gradient. Particles (shown in orange) extravasate into extracellular space where they are collected in regions of high magnetic field gradient. Reproduced with permission from Al-Jamal K.T., Nano Letters; published by American Chemical Society, 2016 (**B**) Applied single magnets only pull in one direction towards the magnet versus dual magnets that can maintain a more constant gradient resulting in a constant outward radial force. (**C**) Magnetic set-up from Liu et al. using two oppositely polarized magnets to enhance magnetic drug targeting in deep tis-sues. Current methods use a single applied magnet resulting in limited use to surface level depths compared to dual magnets. The magnetic gradient of a single magnet falls off very quickly as distance increases compared to the pro-posed dual magnet device, which maintains the magnetic field with an increase in distance. Reproduced with permission from Liu et al., ACS Nano; published by American Chemical Society, 2020 Modeled using art modified from Servier Medical Art, licensed under a Creative Common Attribution 3.0 Generic License, http://smart.servier.com/ (accessed on 20 April 2021).

This modest targeting performance could be vastly exceeded with magnetic drug delivery systems. Early investigators envisioned applied magnetic fields that were positioned around an organism capturing magnetic nanoparticles within tissue (Figure 2) [14,38]. As an example, an intravenous injection of a magnetic nanoparticles yields bloodborne particles that could be captured or collected in a solid tumor that was subjected to large magnetic field gradients. Such gradients could be generated by a magnetic system external to the animal or by permanent magnets inserted into the target tissue. The reliance on the physical separation of magnetic materials within a biological system for targeting delivery is a fundamentally different approach to targeting than the chemical and biological strategies that were introduced earlier. If successful, this approach could increase the efficacy of delivery, limit off-target effects, and reduce the overall amount and time course of treatments [39].

Magnetic nanoparticles that have been explored for targeted drug delivery have had to meet many stringent demands. Their dimensions and surface treatments must balance particle circulation time, drug distribution, drug release, accumulation, and, if needed, cellular uptake [25]. For most exposure routes (e.g., intravenous, oral, etc.), investigators aim for hydrodynamic diameters between 10 and 200 nm [37]. The application of polyethylene glycol (PEG) as a surface coating can prolong the circulation of intravenously injected materials, even with some degree of targeting functionality [40]. Iron oxide-based magnetic nanomaterials are of particular interest, because various SPION formulations have been approved by the US Food and Drug Administration (FDA) for various applications, including as MRI contrast agents [41]. While these materials are not widely adopted by radiologists due to the difficulty in interpreting T_2_ contrast signals, they have found success off-label as treatments for iron deficiency [42]. Other challenges for the clinical translation of magnetic drug delivery systems include the reproducibility and scale of particle production, the economic feasibility of the application, and the practicality and safety of effective external magnetic field application. Magnetic drug delivery is also limited by the fact that particles are not retained at a target site once the external field is removed, which precludes many longer and chronic drug delivery applications [43].

In addition to tackling these clinical obstacles, investigators are also broadening the appeal and reach of magnetic drug delivery [9,44,45]. One avenue of exploration is to increase the benefits of magnetic drug delivery through the integration of multiple delivery and imaging modalities. For example, Hervault et al. developed magnetic nanocomposites (MNCs) that included both a hyperthermic agent as well as a drug carrier for applications of multimodal cancer therapy [44]. By combining pH and thermo-responsive behavior, they could spatially and temporally control the release of Doxorubicin, which is a common chemotherapeutic agent. Chen et al. demonstrate that multifunctional envelope-type mesoporous silica nanoparticles (MEMSN) can increase the specificity of drug delivery and enhance the contrast of magnetic resonance imaging (MRI) [45]. This is achieved through a release system that is initiated in acidic environments via the reactivity of immobilized surface acetals. This acid-catalyzed surface coating results in burst release of the target drug, Doxorubicin, in the slightly acidic tumor microenvironment allowing for efficient and targeted delivery of an otherwise highly toxic anticancer therapeutic. When addressing the treatment of glioblastoma, specifically with Doxorubicin, passage through the blood brain barrier has to be considered. Norouzi et al. developed a Doxorubicin loaded magnetic combination therapy that displayed a dramatic increase in passage through the blood brain barrier. This 2.8 fold increase is due to the use of cadherin binding peptides, which transiently open the tight junctions of the blood brain barrier, combined with the use of an external magnetic field to draw the particles to the target region [46]. This work, like many others in the field, shows the promising impactful change that magnetic combination therapies can have.

Dual drug delivery and imaging nanoscale delivery systems, which are often termed theranostics, can be useful for both therapeutic and diagnostic purposes. Luque-Michel et al. developed theranostic polymeric nanoparticles loaded with SPIONs and doxorubicin to treat glioma-bearing mice [47]. They found significant particle accumulation when the animal is under static magnetic field and the accumulation was easily imaged using MRI. Theranostics are the logical next step for magnetic nanoparticle applications, since the same material can be used in multiple ways. Currently, researchers are forming hybrid magnetic nanoparticles to optimize the optical or chemical properties. This can be the addition of gold, manganese, sulfides of copper, or tungsten, which increases the particles’ magnetism and relaxivity whrn compared with non-doped SPIONS [48]. By combining different material characteristics, more effective and less toxic theranostics can be developed.

Magnetic gene delivery is also of ongoing interest to researchers because of its broad significance. Often referred to as magnetofection, this type of magnetic drug delivery attaches magnetic carriers to a viral vector carrying a therapeutic gene [11]; in some cases, more rarely, the nucleic acid is directly linked to a magnetic nanoparticle via ionic interactions. In 2002, Scherer et al. presented the first example of magnetofection in vitro and demonstrated that transfection efficiency could be increased by the application of a localized external magnetic field [49]. Nearly two decades later, research into magnetofection is focused on reducing the time for magnetic transfection, minimizing the vector dose, and expanding gene delivery to in vivo transfection in lung epithelium and blood vessel endothelial cells [50,51,52]. The current challenges facing application of this delivery system in vivo are the potential for magnetic nanoparticle agglomeration and poor transfection efficiency if the viral carrier is removed [51]. Indeed, magnetofection has high transfection efficiency when compared to other methods, and it is a commonly used technique for in vitro applications.

Finally, any use of external magnetic fields to manipulate particles in vivo requires efficient systems for applying them. Until recently, single electromagnetic coils or permanent magnets were used for this purpose. Clinical applications would require much larger magnetics, increasing power demands, the need for efficient cooling systems, and cost. Originally, large magnetic field gradients generated inside of electromagnetic coils directed magnetic particle movement, but only towards the magnet instead of holding them at the region of interest. Nacev et al. used multiple focusing magnets to address this issue and to extend the reach of external fields to areas that are deeper within the body [53]. They used fast magnetic pulses to trap ferromagnetic rods at specific locations, resulting in inward-pointing magnetic forces. These forces were, in effect, focused, and lead to a larger field gradient and more specific and localized targeting. Although they did not apply their methodology to drug delivery, this more specific and targeted approach has the potential to overcome some of the largest barriers to entry for clinical applications. In another example, Liu et al. positioned permanent magnets in an opposing square (a simplified model is shown in Figure 2C) to improve the accumulation and penetration of magnetic nanocarriers into solid tumors [54]. They demonstrated a five-fold increase of penetration and a three-fold increase in the accumulation of magnetic nanoparticles when compared to passive accumulation alone. Moreover, the system could reach deeper into tissue than approaches that rely on a single permanent magnet that can only collect materials at superficial depths, typically only a few millimeters for a rare earth permanent magnet. This two-magnet configuration is just one example of emerging magnet designs that improve the efficacy, accumulation, and movement control of magnetic nanoparticles, bringing magnetically driven drug targeting closer to the clinic.

### 2.3. Magnetothermal Heating

The magnetothermal heating of magnetic particles was first observed in 1954, where it was used to selectively destroy cancer metastases in lymph nodes that might have been previously missed in surgery [55]. Briefly, magnetothermal heating occurs when magnetic particles are subjected to alternating magnetic fields (AMFs). Through magnetic induction, nanoparticles in AFMs are selectively heated, providing for localized increases in temperature. The effect can be used in drug delivery schemes that apply thermally sensitive coatings to nanoparticles, which result in the release of chemotherapeutic agents in addition to the thermal ablation of the cancer cells [56,57]. Magnetothermal treatments have been approved in the European Union (EU), and they were also approved by the US Food and Drug Administration (FDA) in 2006 for phase I clinical trials in the treatment of prostate cancer. Ongoing clinical applications have been limited by the need for precise placement of large AMFs within the human body [8]. Conventionally, the organism is placed within an electromagnetic coil, but this can be difficult with larger animals. The duration of heat treatment and the strength of the AMFs are also important parameters to control with existing methods.

Magnetothermal heating can be very heterogeneous, leading to insufficient and unpredictable heating, because of tumor vasculature and extracellular matrix structure. Silva et al. combined magnetic nanoparticles with green fluorescent protein to form “nanothermometers” that use feedback to minimize heterogenous heating [58]. While the early days of magnetothermal heating were concerned with heating tissue to high temperatures (>45 °C) to kill cells, recent interest has centered on using mild heating to influence biological processes with great precision. Christiansen et al. used the localized heating of magnetic nanoparticles to actuate neuronal ion channels from a distance using magnetic nanoparticles [56]. Other researchers have also used AMF heating to open and close an ion channel without affecting the health of cells [12]. Radio-frequency magnetic fields can also remotely activate cation channels in cells deep within tissue, thereby offering an alternative to the limited depth penetration of photothermal therapies. However, a more recent trend attempts to pair photothermal and magnetothermal together to give a secondary “activation” force to carry out the necessary heating even deeper within the body for applications from arterial inflammation to cancer therapies [34,59]. This combination therapy is ten times more effective at heating the target region than the individual use of these therapies [60]. This combination of photothermal and magnetothermal therapies can be used to apply hyperthermia treatment and release drug to the region of interest. This is demonstrated by Lu et al. and their work with modified iron oxide composite nanoparticles loaded with cetuximab. Combination thermal heating was used for both applying hyperthermia treatment and to thermally release drug [61].

However, more stable and sensitive magnetic particles are needed to make the clinical translation of magnetothermal therapy more feasible. Some investigators have also reported challenges with superparamagnetic iron oxide nanocrystals (SPION) aggregation. Therefore, without proper surface engineering, the use of SPION in magnetothermal applications like tumor treatment could be limited [62]. More recently, these challenges are being met in a variety of ways, and several recent review papers cover these advances with respect to magnetothermal heating [28,32,34,36]. The responsiveness of magnetic particles to smaller AMFs can be optimized by altering their composition and shape to increase their magnetic susceptibility [28]. Doped ferrites are a promising approach for increasing susceptibility, and therefore sensitivity, without complicating their surface engineering [63]. Different nanoparticle shapes, such as the magnetic nanoplates proposed by Alhasan et al., allow for more efficient heating with lower AMFs [62].

## 3. Imaging

### 3.1. Magnetic Resonance Imaging (MRI) Contrast Agents

A common medical application for magnetic nanoparticles is their use as contrast agents for magnetic resonance imaging (MRI). MRI is a non-invasive and high-resolution imaging modality that has become the clinical standard for visualizing anatomical structures. In spite of its wide clinical use, MRI has low signal intensity and sensitivity, which makes rapid and accurate diagnoses difficult [64]. Consequently, approximately 40–50% of MRI procedures require contrast agents for image enhancement [65]. Gadolinium chelates (GCs) are the current clinical standard for MRI because of their low toxicity, short circulation half-life, and positive contrast enhancement [7,66,67]. However, concerns have been raised regarding potential toxicity, non-specific biodistribution, poor cellular uptake and retention, and the sub-optimal contrast enhancement of GCs [7,68,69]. As a result, many improvements and alternatives to GCs have been developed [7,70,71,72,73,74,75,76,77].

Being developed as gadolinium-free alternatives to GCs, iron oxide particles (IOP) garnered clinical interest as MRI contrast agents because of their useful magnetic properties, unique biodistribution and pharmacokinetic profiles, targeting potential, and biocompatibility [78]. Early successes with superparamagnetic iron oxide nanocrystals (SPIONs, D_H_ > 50 nm) and ultrasmall SPIONs (USPIONs, D_H_ < 50 nm) led to the development of IOP with more robust synthetic approaches and a range of physiochemical, magnetic, biodistribution, and pharmacokinetic properties (Table 1). These materials have demonstrated preclinical and clinical potential, but many have been commercially discontinued for MRI and are only used in non-MRI clinical applications (Table 1).

The notable failure of iron oxide particles (IOP) to become standard tools in clinical MRI is generally ascribed to two distinct challenges. First is the reluctance of healthcare providers to use IOP in their regular practice. This is due, in part, to toxicity concerns that are amplified by black box warnings issued by the US Food and Drug Administration (FDA) after studies showed small, but measurable, risks of serious adverse events (0–1%) and anaphylaxis (0.02–0.2%) after ferumoxytol administration [66,85]. Additionally, radiologists are not as experienced in interpreting the dark contrast provided by IOP in transverse water relaxation time (T_2_)-enhanced MR images [85,86]. Dark contrast enhancement and susceptibility artifacts from IOP can result in misdiagnosis and an overestimation of lesion margins [70,85,86,87]. A second issue has been the reluctance of pharmaceutical companies to produce IOP contrast agents. The demand for IOP is low because of healthcare provider hesitancy, niche application (e.g., liver-, spleen-, and lymph node-related imaging and patients with renal deficiency), and ongoing concerns regarding their diagnostic utility when compared to conventional contrast agents [88,89,90].

In response to these issues, researchers have continued to develop IOP to reduce toxicity concerns, optimize magnetic properties and contrast performance, and apply them in novel and significant ways [65,66,72,73,74,75,86,88]. Here, we focus on the latter, and examine the current trends in IOP-based MRI.

**Figure 3:**
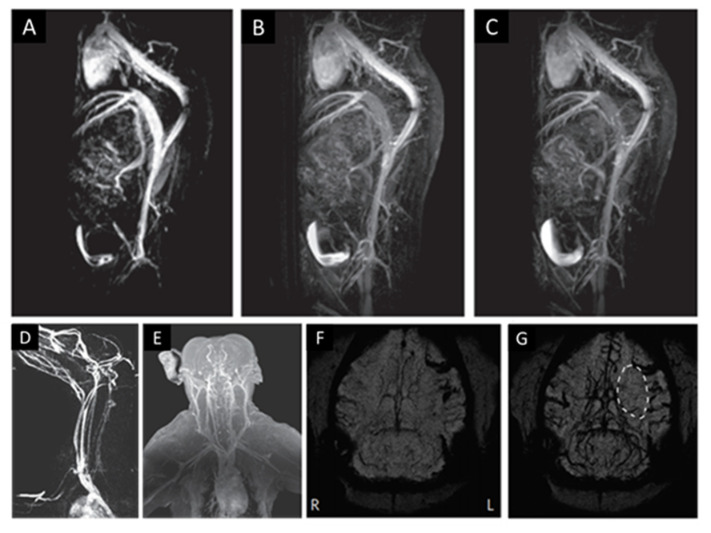
T_1_-weighed MRA of a mouse at (**A**) 4, (**B**) 12, and (**C**) 20 min post injection with ZES-SPIONs. MRA of (**D**) canine (beagle) and (**E**) non-human primate (macaque) animal models post PEG-IONC injection. Dynamic susceptibility contrast perfusion-weighted images of left cerebral ischemia in a macaque (**F**) before and (**G**) after bolus injection of PEG-IONC. (**A**–**C**) Reproduced with permission from Wei et al., Proceedings of the National Academy of Sciences of the United States of America; published by National Academy of Science, 2017. (**D**–**G**) Reproduced with permission from Lu Y. et al., Nature Biomedical Engineering; published by Springer Nature, 2017.

IOP have been mostly relegated to mononuclear phagocyte system (MPS)-related imaging (e.g., liver, spleen, and lymph nodes) and cellular tracking applications [70]. To overcome radiologists’ concerns about the dark contrast resulting from T_2_ manipulation, IOPs are being developed as longitudinal water relaxation time (T_1_) contrast agents [86,88]. This provides the desirable white contrast in images, and T_1_ enhanced magnetic nanoparticles are typically smaller, and they yield greater signal-to-noise (tissue T_1_ > T_2_) and better spatial resolution than those developed for T_2_ applications. This makes the materials relevant for a wider variety of applications. For instance, Wei et al. developed a zwitterion-coated exceedingly small SPION (ZES-SPION, D_H_ = 4.7 nm) for magnetic resonance angiography (MRA) in small animals (Figure 3A–C) [87]. These ZES-SPIONs are biocompatible, renally cleared (unlike commercial USPION), and possess T_1_ contrast and blood circulation times that are comparable to commercial GCs [67,87]. Lu et al. used slightly larger polyethylene glycol (PEG)-coated USPIONs (PEG-IONC, D_H_ = ~12 nm) to study the toxicity and potential of IOP as T_1_ MRI contrast agents in larger animal models (Figure 3D–G) [89]. PEG-IONCs demonstrated no significant toxicity and they were successfully used for full-body MRA; notably they were able to identify ischemia in cerebral angiograms. More recently, Kang et al. used similar USPION in rats to monitor the remodeling of cerebral vasculature after ischemic stroke [91].

**Figure 4:**
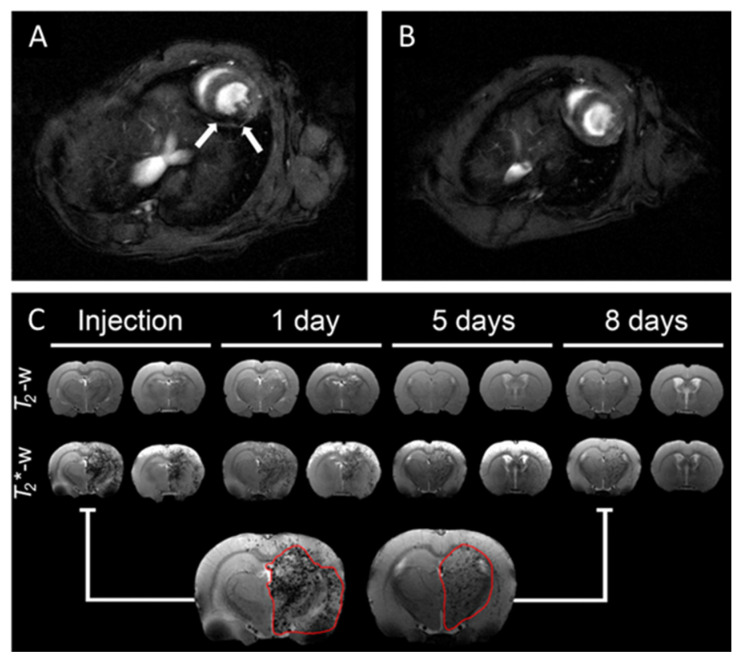
T_2_-weighted MR image of mouse (**A**) without and (**B**) with intracardially implanted SPION-labeled myoblasts. (**C**) T_2_/T_2_*-weighted cerebral MR images of mice intra-arterially injected with USPIO-PAA-GlcN-labeled mesenchymal stem cells after 1 h, 24 h, 5 days, and 8 days. (**A**,**B**) Reproduced with permission from Wierzbinski, K. R. et al., Scientific Reports; published by Nature Research, 2018. (**C**) Reproduced with from Guldris, N. et al., Bioconjugate Chemistry; published by American Chemical Society, 2017.

Cellular tracking and labeling are another common trend in preclinical and clinical IOP-based MRI [80,91]. Because T_1_ imaging can be significantly impacted by compartmentalization of nanoparticles in cells, applications usually use T_2_-weighted MRI [66]. Guldris et al. developed glucosamine-modified polyacrylic acid-coated USPIONs (USPIO-PAA-GlcN, D_H_ = 40 nm) for enhanced cellular uptake and biocompatibility, and use in long-term MRI tracking of intra-arterially injected stems cells in healthy rat brains (Figure 4C) [92]. When compared to PAA-coated SPIONs and USPIONs, USPIO-PAA-GlcN demonstrate greater promise for potential in vivo applications in tracking the stem cell treatment of cerebral ischemia. However, there are concerns that IOP can adversely impact the functions of labeled cells [85,93]. Wierzbinski et al. labeled human skeletal myoblasts with carboxylic acid-coated USPION (DMSA-SPION, core size = ~10 nm) to track integration after implantation into the left heart ventricle of mice (Figure 4A,B) [94]. DMSA-SPIONs had no significant functional or cytotoxic effect on myoblasts. Moreover, the work demonstrated the potential for clinically tracking the integration and progress of skeletal myoblast transplants into postinfarction scars. Ultimately, the adverse effects on labeled cells can be reduced with more biocompatible and responsive IOP to enable a lower effective nanoparticle dose.

**Figure 5:**
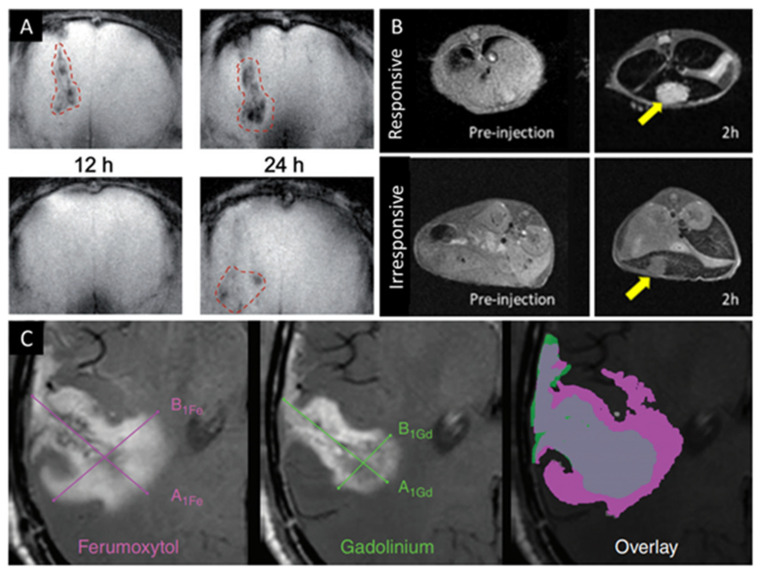
(**A**) T2-weighted MR images of a U-87 MG (human glioblastoma) tumor in the brain of a nude mouse using iron oxide nanocubes (top) with B6 peptide and (bottom) without at 12 and 24 h intervals after intravenous injection. (**B**) T1-weighted MR images of orthotopic hepatocellular carcinoma mouse models before and 2 h after injection with pH-responsive (top) and pH-irresponsive (bottom) USPION clusters. (**C**) Representative ferumoxytol- and GC-enhanced MR images of a patient with glioblastoma and an overlay of the two demonstrating the mismatch used to distinguish between pseudoprogression and true progression. (**A**) Reproduced with permission from Lu, Z. et al., Advanced Functional Materials; published by John Wiley and Sons; 2017. (**B**) Reproduced with permission from Lu, J. et al., Journal of the American Chemical Society; published by American Chemical Society, 2018. (**C**) Reproduced with permission from Barajas, R. F. et al., Neuro-Oncology; published by Oxford University Press, 2019.

IOP are also being used in a wide variety of passive and active targeting-based molecular MRI applications [64]. Sherwood et al. developed bovine serum albumin (BSA)-USPION clusters (core sizes <4 nm, cluster size = ~200 nm) for MR image-guided drug delivery to subcutaneous tumor-bearing mice [95]. This is possible because tumors often exhibit molecular features that can cause porous vasculature and poor lymphatic drainage, which results in the passive accumulation of nanoscale materials—often called the enhanced permeability and retention (EPR) effect [96,97]. Others have developed pH responsive USPION clusters to take advantage of, and target, the slightly lower pH (pH 5.6–6.8) of the tumor microenvironment [98,99]. In the presence of the slightly acidic tumor microenvironment, pH-sensitive cluster crosslinkers disassociate, causing the release of smaller USPION, which allows for greater accumulation, signal-to-noise, and T_1_ contrast enhancement (Figure 5B). IOP contrast agents can also be used for the molecular imaging of the inflammation that is associated with pain because of the greater presence of MPS cells—which preferentially uptake foreign nanoscale objects [67]. A few recent clinical studies highlight the advantages of molecular imaging by comparing USPION- and GC-enhanced MRI for assessing a variety of disease states that are associated with inflammation as well as tumors [86,87,100,101]. In all cases, T_1_- and or T_2_-weighted USPION-enhanced MRI provided equal or greater diagnostic utility when used alone or in conjunction with T_1_-weighted GC-enhanced MRI. Notably, Barajas et al. demonstrated that dual ferumoxytol- and GC-enhanced MRI could reliably differentiate between true progression (recurrence) and pseudoprogression (therapy-associated tissue damage and inflammation) by observing biodistribution-associated mismatch in their imaging enhancement (Figure 5C) [102].

In response to critiques of EPR-based passive accumulation, actively targeted IOP are being used to further increase the specificity and sensitivity of molecular MRI [96,103]. Because transferrin receptors (TfR) are overexpressed in glioma, Lu et al. attached a TfR-specific peptide (B6) to a SPION-based drug delivery system (CARD-B6) for targeted T_2_ imaging of glioma [104]. When compared to non-targeted CARD, CARD-B6 demonstrated much greater accumulation inside the tumor margins (Figure 5A). Husain et al. targeted excess matrix metalloproteinase (MMP-12) that was associated with inflammation to image molecular features associated with neuropathic pain in rats [100]. Even with these IOP-based molecular MRI techniques, sensitivity is a concern, because accumulation can often be too low to achieve meaningful contrast enhancement [85]. Current efforts focus on enhancing the magnetic properties of IOP to decrease the effective dose, reducing the associated toxicity and imaging artifacts [5,73,101].

### 3.2. Magnetic Particle Imaging (MPI) Tracers

Magnetic particle imaging (MPI) is a novel imaging technique that was first proposed in 2001 [105]. MPI detects signals from superparamagnetic nanomaterials, also referred to as MPI tracers, which are generated by a fast-moving magnetic field-free region (FFR) [105,106]. In 2005, Gleich et al. demonstrated that this signal can be processed to reflect tracer spatial location and concentration, thereby offering an opportunity for quantitative imaging with high spatial resolution (~1 mm) and sensitivity (~100 µmol Fe/L) [107]. Additionally, since superparamagnetic tracers are not naturally present in the body, MPI has nearly zero background, as compared to the clinical contrast-enhanced MRI. Following the development of early preclinical prototypes in the late 2000s, Weizenecker et al. performed the first in vivo three-dimensional MPI experiment examining the beating heart of a mouse in real-time [105,108]. Despite this success, the clinical translation of MPI depends on the development of much larger scanners and highly responsive tracers to further enhance spatial resolution and sensitivity [109,110,111,112]. MPI tracer performance is dependent on its ability to reverse its magnetic moment in the FFR; the larger the change in magnetic moment, the larger the MPI signal. As with any nanomedicine, the colloidal stability, pharmacokinetics, biodistribution, and biocompatibility of the magnetic nanoparticles for MPI are also important considerations.

**Figure 6:**
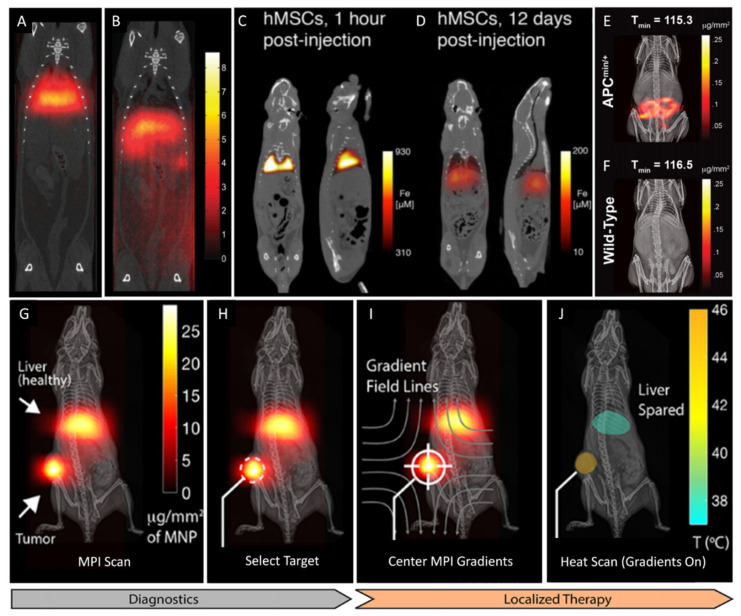
MPI of intravenously administered (**A**) MAA-SPION and (**B**) SPION in healthy rats. The larger MAA-SPION target the lungs while smaller SPION distribute primarily to the liver. MPI of tracer labeled human MSCs (**C**) 1 h and (**D**) 12 days after intravenous administration in healthy rats. Labeled MSCs move from the lungs to the liver and spleen over the course of 12 days. The subtraction MPI of (**E**) GI polyp/bleed and (**F**) normal mouse models about 2 h post intravenous administration. Signal evident in the intestines for mice with GI bleed. (**G**–**J**) Procedure for MPI-guided localization and magnetic hyperthermia therapy. The diagnostic stage involves (**G**) the initial MPI scan of the tumor-bearing mouse model and (**H**) selecting the target. The localized therapy stage involves (**I**) centering the FFR on the target followed by (**J**) the therapeutic heat scan. (**A**,**B**) Reproduced with permission from Zhou, X. Y. et al., Physics in Medicine & Biology; published by Institute of Physics and Engineering in Medicince, 2017. (**C**,**D**) Reproduced with permission from Zheng, B. et al., Theranostics; published by Ivyspring International Publisher, 2016. (**E**,**F**) Reproduced with permission from Yu, E. Y., et al., ACS Nano; American Chemical Society, 2017. (**G**–**J**) Reproduced with permission from Tay, Z. W., et al., Nano Letters; published by American Chemical Society, 2018.

As tracer technology continues to develop, MPI can be applied in a wide range of biomedical applications [109]. Zhou et al. performed the first in vivo MPI of lung perfusion in rats (Figure 6A,B) [110]. Here, micron-sized bovine serum albumin (BSA)-conjugated SPION aggregates (MAA-SPION, ~25 µm) were used to target the narrow capillary bed of the lungs (6 µm) after their first pass through the heart. When compared to standard diagnostic techniques for assessing pulmonary embolism, this preliminary study on healthy rats demonstrates the potential of MAA-SPION-based MPI as a convenient and ionizing radiation-free alternative to other diagnostic options. The first-pass pulmonary trapping of micron-sized objects, while useful for lung imaging, presents a problem for the intravenous therapeutic delivery of mesenchymal stem cells (MSC). To better understand the biological fate of cellular therapies, Zheng et al. used quantitative MPI to assess the biodistribution and pharmacokinetics of tracer-tagged MSCs (Figure 6C,D) [113]. MPI can also be used to visualize and assess disease states. For instance, Yu et al. used subtraction MPI to quantify the extent of gastrointestinal (GI) bleeding in a mouse model that was predisposed to developing GI polyps (Figure 6E,F) [114]. MPI offers a non-invasive, non-ionizing, and rapidly administered alternative when compared to traditional approaches for assessing GI bleeds (e.g., colonoscopy and radionuclide scintigraphy). As with magnetic nanoparticle magnetic resonance imaging (MRI) contrast agents, MPI tracers can also take advantage of the enhanced permeability and retention (EPR) effect and passive accumulation to image tumors when possible [111].

Apart from simple tumor imaging, MPI can be used for therapeutic purposes. For example, Zhu et al. used quantitative MPI to monitor in vivo drug release in tumor-bearing mice [112]. Their unique MPI tracer is a pH-sensitive SPION-drug cluster that, when introduced to the acidic tumor microenvironment, releases SPION and doxorubicin. Increased SPION Brownian motion after release enhances the MPI signal, and it provides an indirect, but accurate, measure of drug release. Likewise, Tay et al. used SPION tracers for MPI-guided magnetic hyperthermia therapy on a tumor bearing mouse (Figure 6G–J) [115]. MPI is used to map the distribution of SPION, the FFR is moved to the region of interest (tumor), and a second alternating magnetic field is then applied for magnetic hyperthermia in that region only. The ability to precisely monitor the location and magnitude of therapy applied (e.g., drug release or magnetic hyperthermia) would allow for more accurate dosing and tracking of therapeutic efficacy, thus optimizing treatments.

## 4. Movement

### 4.1. Cell Separation

**Figure 7:**
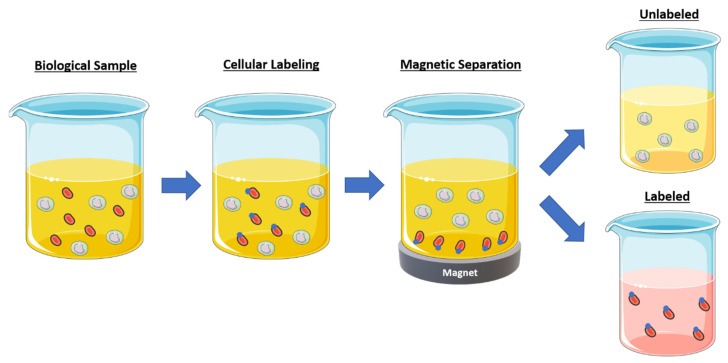
Magnetic batch separation for cell separation. Initially cells are suspended and then the desired cell population is labeled with magnetic nanoparticles. The final step depends on the selection methodology: labeled or unlabeled selection. In unlabeled selection, the desired cells remain in the supernatant and the labeled cells are magnetically captured via a permanent magnet (also known as negative selection). Alternatively, the cells of interest can be labeled and magnetically captured, and the supernatant can be discarded (also known as positive selection). Art modified from Servier Medical Art, licensed under a Creative Common Attribution 3.0 Generic License, http://smart.servier.com/ (accessed on 20 April 2021).

The magnetic separation of biological material using particles was first applied in the 1970s to sorting cells [116] and, since then, “magnetophoresis”, as it has been termed, is widely used to separate specific cells from a biofluid or trim down cell populations (Figure 7) [117]. The speed and ability to batch process biological samples make magnetic-activated cell sorting (MACS) an especially appealing option for cell sorting in flow cytometry instruments [117]. The current limitations of magnetic separation for this application include high sample processing cost, limited sample throughput, low processing speeds, and loss of cellular function or viability [117]. However, magnetophoresis in the scaled-down environment of microfluidic systems faces fewer of these issues and remains an expanding area of research.

One area of focus for research in this area has been single cell capture as it relates to cancer diagnostics. The internal capture of circulating tumor cells, for example, is possible using an intravascular magnetic wire implanted into a patient, and magnetic particles offer less invasive, but similar, opportunities [118]. External use of microfluidics, often termed “lab on a chip”, can be applied to the analysis of small drops of biofluids in which magnetic nanoparticles can be used to separate cells using antibodies or proteins as markers [119,120,121,122,123,124]. Alternatively, Robert et al. was able to sort monocytes and macrophages by exploiting the different internalization rates of iron oxide nanoparticles [125]. The macrophages were sorted into five different groups, depending on the nanoparticle load using on-chip free-flow magnetophoresis. Monocytes had a much lower capacity to internalize particles and, as a result, were far less magnetic, thereby providing an excellent on-chip example of negative selection. Zhang Q. et al. demonstrated an immuno-magnetic sorting procedure using four types of immuno-magnetic nanoparticles for the separation of different T cells [126]. They found that selectivity could be preserved, even at processing volumes as high as four liters of processed blood sample, but noted that increased throughput did degrade the selectivity of the separation process. While many examples of magnetic cell-sorting have been developed for the research laboratory, there is some promise that the technology could be relevant to consumers. Tran et al. demonstrated a supraparticle assembly of magnetic nanoparticles for selective cell separation and counting using a smartphone-based imaging platform [127]. The integration of magnetic particles with “lab on a chip” technology has been advantageous in many biomedical applications.

### 4.2. Soft Robotics

**Figure 8:**
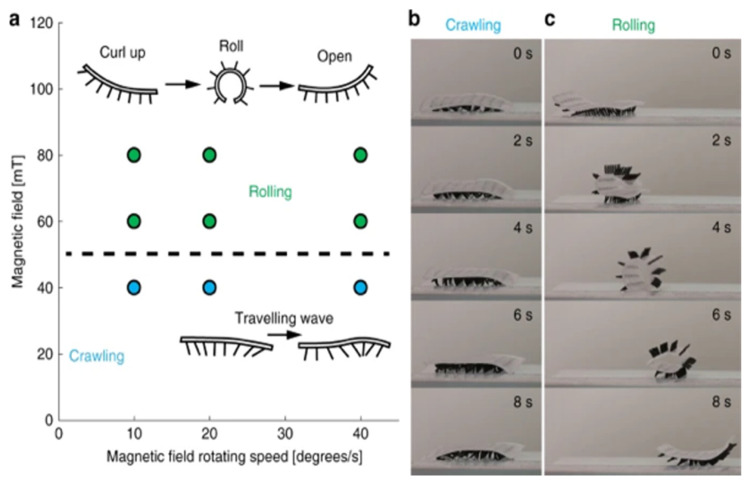
(**a**) Work from Gu H. et al. displays different modes of locomotion possible using magnetically actuated cillia including crawling and rolling. (**b**) Metachronal waves of the cilia structures leads to a crawling motion (**c**) When the magnetic field is larger than 60 mT the strong magnetic torque leads the soft robot to roll. Reprinted without changes with permission through the Creative Commons License 4.0 International License from Gu H. et al., Nature Communication; published by Springer Nature Limited, 2020.

Soft robotics is one of the most novel applications of magnetic nanoparticles in the field of directed motion. Soft robotics refers to systems that are built with flexible and stretchable materials to mimic living, moving tissue [128]. Being inspired by natural systems, nanoparticles can be incorporated into soft robotics to facilitate actuation of movement on a macro-scale and, if biocompatible, demonstrate promise for biomedical applications. Soft robots have been introduced into surgery, diagnosis, drug delivery, wearable and assistive devices, prostheses, and even artificial organs [129]. Most soft robots are quite large—on the order of millimeters—and their movement mechanisms are often electrically actuated. Magnetically actuated microrobots, while being more difficult to design, are of great interest, as they can be controlled at a distance without the need for a connection to a power source [130]. Magnetic microrobots that are subjected to applied magnetic fields can exhibit a wide range of deformations allowing for multiple types of movement, including rolling, walking, crawling, and jumping [131]. Magneto-elastic soft millimeter-scale robots offer greater movement due to their higher degrees of mobility, and they have been even shown to be able to transit between different liquid and solid terrains as well as switching between different locomotive modes. Although not at the nanoscale, Gu et al. developed magneto-elastic microrobots that mimic natural cilia—the hair-like structures that are found on microorganisms. The programmable robots can generate metachronal waves, making them able to crawl and roll, depending on the strength of the magnetic field, as seen in Figure 8 [132].

The limitations of current magnetically-actuated soft robots include their difficulty navigating unknown obstacles, poor response to environmental change, and large millimeter sizes that limit clinical application [132]. Iron oxide nanoparticles can be incorporated into elastomeric matrices that can be shaped into sub-micron objects to reduce the size of these soft robots. Bayaniahangar et al. 3D printed helical coils using a ferrofluid-siloxane mixture that could be actuated via external magnetic field [133]. Gouda et al. and Breger et al. created “micro-grippers” by embedding superparamagnetic iron oxide nanocrystals (SPIONs) into biodegradable matrices, so that the programmable 3D structures could be non-invasively triggered via external field. These magnetic structures were biodegradable, thereby eliminating the need for a second surgery for removal [134,135]. Hwang et al. demonstrated that multifunctional soft robots responsive to external magnetic fields can efficiently, and precisely, destroy biofilms. They built catalytic antimicrobial robots (CARs) that generate bactericidal free radicals that break down biofilms, and then remove the fragmented biofilm via magnetically directed processes. Such concepts may find applications in areas that range from wound care to dentistry [136]. Current trends focus on increasing the magnetic sensitivity of the embedded particles as well as exploring the wide space of combined chemical and mechanical activity [116,137,138].

## 5. Diagnostics

### Immunoassays

**Figure 9:**
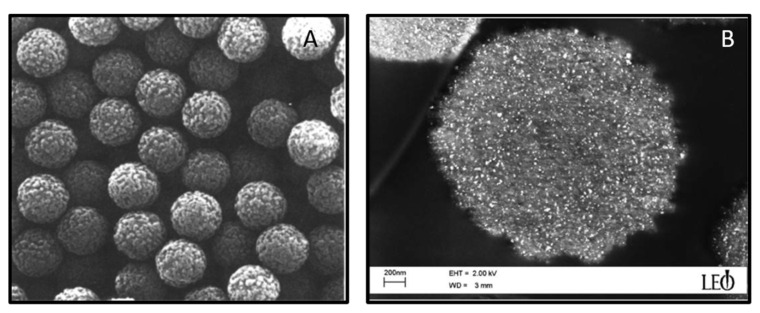
(**A**) Polystyrene beads of average diameter 2.8 microns containing 12 *w/w*% iron in their pores. (**B**) SEM of a M-280 bead from Dynabeads^TM^. The nanoparticles in the bead are visualized as bright points and were determined to be ~8 nm in diameter. (**B**) Reproduced with permission from Ugelstad et al., Progress in Polymer Science; published by Elsevier, 1992. Reproduced with permission from Fonnumm et al., Journal of Magnetism and Magnetic Materials; published by Elsevier, 2005.

The attraction of magnetic nanoparticles towards externally applied fields is the basis of their use for diverse biological detection problems. Research in this area dates back to 1976, when a Norwegian scientist, John Ugelstad, exploring the synthesis of uniform polymer spheres for chromatography, first precipitated iron oxide nanoparticles into the porous colloids [139]. This yielded polymer particles, typically 20–30 *w/w*% iron oxide, which could be readily captured via rare earth, handheld magnets. Later research revealed that the materials were nanoscale maghemite, superparamagnetic, and well dispersed throughout the micron-sized polymer beads (Figure 9) [140]. Among their first applications was the treatment of pediatric neuroblastomas in which the magnetic beads were used to separate tumor cells from patient’s bone marrow prior to autologous transplantation [141,142]. By decorating the surface of the particles with an antibody to known tumor cell antigens, investigators found that they could reduce the population of tumor cells in aspirates by three orders of magnitude. Through appropriate surface design, researchers throughout the early 1990s extended this flexible platform beyond cell-based separations to include the isolation and detection of proteins, nucleic acids, viruses, and bacteria [143,144,145].

Commercial entities quickly capitalized on these magnetic beads for applications in biomedical research enabling the development of clinical applications. Such effort required reliable and reproducible materials and companies, such Dynabeads^TM^, were able to meet the need for high quality nanoparticles. By 1996, there was a robust commercial business that provided researchers with magnetic beads, in both small (1 µm) and large (2.5 µm) diameter formats, with an array of different surface coatings. Biomedical researchers used benchtop magnetic separators and these beads as alternatives to tedious, multi-step purification protocols for various biomolecules, while clinical researchers began to explore bead-based analysis for disease detection, as described in Section 4.1. In one example, investigators correlated the success of kidney transplantation to the number of circulating epithelial cells that were recovered via immunomagnetic capture [146]. Magnetic beads were also used to analyze the DNA retrieved from patients with meningitis, so as to confirm its bacterial origins [147].

The past five years have seen continued growth in magnetic bead technology for diagnostics, as their application has expanded substantially into the in vitro diagnostics of both protein and nucleic acids. Bead technology, and specifically magnetic beads, are now viewed as an increasingly attractive alternative to the enzyme-linked immunosorbent assay (ELISA) platform. This interest is driven, in part, by the pressing need for automation and simplified sample and liquid handling. Magnetic beads are well suited to such an environment, as they can be held fixed in place while robotic systems introduce reagents and eluent buffers. Several companies now sell commercial versions (MagPix^TM^) of systems that utilize these advantages, and the immunoassays perform at least as well, or even better, than the conventional ELISA systems [148,149]. The simplified handling of magnetic particles is also of great value in the preparation of samples for quantitative polymerase chain reaction (qPCR), as was demonstrated in the sensitive detection of Tuberculosis pathogens using a magnetic bead to gather sample DNA (e.g., amplicons) [150].

Multianalyte detection is a major theme in modern clinical diagnostic research, and magnetic beads are poised to play a central role. The rich abundance of proteomic and genomic information now readily available has established a growing need for the simultaneous detection of multiple biomarkers, ideally without extra cost or time. Commercial schemes leverage the capability to form libraries of beads, each being “barcoded” with optically distinct molecular fluorophore signatures, and each tailored with a unique surface targeting different biomolecules. Early versions of this technology used flow-based optical read-out to interrogate non-magnetic beads one-by-one, like conventional flow cytometry [151,152]. The latest systems use magnetic beads that can be draw down into a monolayer; high resolution optical cameras can then image the bead barcodes as well as level of analyte bound over a field. In one case, such multiplex bead-based technology was as effective as sequential ELISA immunoassays for measuring up to ten biomarker proteins for bladder cancer in urine [153]. Also important is the development of magnetic bead-based assays for low resource settings. Paper-based immunoassays using functionalized magnetic beads to replace costly sample preparation steps are the subject of intense study [154]. Such accessible technology is particularly important for the multiplex detection of malaria antibodies for which magnetic bead technology is particularly well suited [155,156,157].

Although commercial magnetic beads are largely unchanged from those applied forty years ago, new magnetic nanoparticles and their composites offer improved performance and new types of applications. Investigators have used ferrites, typically Co-Fe_2_O_4_, instead of iron oxide as a magnetic material, beads are more responsive to applied fields, leading to faster separations [158,159,160]. Control over the dimensions of the magnetic nanoparticles also presents the opportunity to use different field strengths for multiplexed separations. By incorporating gold nanoparticles onto magnetic beads, several investigators have demonstrated more sensitive detection in immunoassays by leveraging particle-generated chemiluminescence or gold particle dissolution [161,162]. Alternatively, immunomagnetic separation events can be confirmed through the precipitation of gold nanoparticles at bead surfaces [163]. Quantum dots can also be incorporated into magnetic nanoparticle composites yielding spectrally encoded beads for multiplexed analysis and have recently been used for malaria detection [164,165].

## 6. Conclusions

The use of magnetism in medicine has come a long way since the days of the ancient Greeks. It is the miniature lodestones of today, magnetic nanoparticles (e.g., SPIONs), which make their dream of healing the human body with magnetic fields a modern reality. SPIONs are unique, in that they are therapeutic agents themselves, through their intrinsic ability to catalyze fenton reactions, but they also have the capacity to deliver specific drugs, gene fragments, or magnetothermal heating to specific areas of interest. Current trends improve this prospective by offering multifunctional particles, more effective magnetic field application systems, and even more magnetically sensitive particles. Researchers working to apply magnetic particles in MRI imaging have been successful in synthesizing SPION contrast agents with no notable toxicity, a higher blood circulation time, and both passive and active targeting capabilities. This new generation of magnetic nanoparticles for both MRI and MPI may ultimately make their use in clinical imaging a reality. Finally, the integration of magnetic particles into “lab on a chip” and other diagnostic settings is both meeting the practical needs for faster and cheaper analysis, while also expanding the possibilities for multiple analyte sensing. Even the emerging area of soft robotics stands to benefit from advances in the magnetic nanomaterials that allow for more responsive and functional systems. Progress in both the development of the magnetic nanoparticles, as well as their expanding biomedical applications, has been swift since Ugelstad’s first report of magnetic polymer particles in 1976. One can only imagine what their continued study over the next four decades will have to offer to both science and medicine.

## Figures and Tables

**Table 1:** Commercial iron oxide particles for clinical magnetic resonance imaging.

IOP Name	IOP Type	Core Size/D_H_ (nm)	r_1_/r_2_ (mM^−1^s^−1^)	B_0_ (T)	t_1/2_ (h)	MRI Applications	Commercial Status	Clinical Approval	References
** Ferristene (Abdoscan) **	MIOP	-/~3500	-	-	oral	GI	discontinued (2000)	-	[7,71,79]
** Ferumoxsil (AMI-121,** ** GastroMARK, Lumirem) **	MIOP	-/300	3.4, 2/3.8, 47	1, 1.5	oral	GI	discontinued (2012)	1996 US/EU (GI MRI)	[7,71,72,79]
** Ferumoxides (AMI-25, Feridex, Endorem) **	SPION	4.5–5.6/50–100	40, ~10/~120–160	0.47, 1.5	2	L, S, BM, CTL, BT	discontinued (2008)	1996 US (L and S MRI)	[7,66,70,71,72,79,80,81,82]
** Ferrixan (SHU 555A, Resovist, Cliavist) **	SPION	~10/60–80	25.4, 9.7/~150–190	1.5	2.4–3.6	L, S, MRA, CTL	available in limited countries	2001 EU/JP/AU (L MRI)	[7,66,70,71,72,79,80,82]
** Ferumoxtran-10 (AMI-227, Combidex, Sinerem) **	USPION	4–6/20–50	23, ~10–20/53, ~65–88	0.47, 1.5	24–36	L, LN, S, MRA, M, CTL, BT	discontinued (2007)	-	[7,66,70,71,72,79,82]
** Ferumoxytol (AMI-7228, Feraheme, Rienso) **	USPION	6.7/20–30	38, 15	0.47, 1.5	10–14	L, LN, MRA, M, I, CTL, BT, BL, S	available	2009 US, 2013 EU (iron deficiency treatment)	[7,66,70,71,72,79,81,83,84]
** Ferucarbotran C (SHU 555C, Supravist) **	USPION	3–5/20–25	24, 10.7/60, 38	0.47, 1.5	6–8	MRA, CTL, M	discontinued	-	[66,70,71,72,79]
** Feruglose (NC100150, PEG-feron, Clariscan) **	USPION	5–7/11–15	20	0.5	2–6	L, LN, P, MRA	discontinued (early 2000s)	-	[7,66,70,71,72,79,82]
** VSOP-C184 **	USPION	4–5	20.1, 14	0.94, 1.5	0.6–1.3	L, MRA, CTL, M	stopped development	-	[7,70,71,72,79]

Abbreviations: iron oxide particle (IOP), micron-sized iron oxide particle (MIOP), superparamagnetic iron oxide nanocrystal (SPION), ultrasmall superparamagnetic iron oxide nanocrystal (USPION), hydrodynamic diameter (D_H_), longitudinal water relaxivity (r_1_), transverse water relaxivity (r_2_), external magnetic field strength (B_0_), circulation half-life (t_1/2_), magnetic resonance imaging (MRI), United States (US), European Union (EU), Japan (JP), Australia (AU), gastrointestinal (GI), liver (L), spleen (S), magnetic resonance angiography (MRA), bone marrow (BM), lymph node (LN), macrophage (M), cell tracking and labeling (CTL), perfusion (P), brain tumor (BT), inflammation (I), sarcoma (S), and brain lesions (BL).

## Data Availability

Not applicable.

## Abbreviations

Magnetic resonance imaging (MRI); US Food and Drug Administration (FDA); magnetic particle imaging (MPI); external magnetic field strength (B_0_); intrauterine devices (IUD); superparamagnetic iron oxide nanocrystals (SPION); reactive oxygen species (ROS); polyethylene glycol (PEG); magnetic nanocomposites (MNC); multifunctional envelope-type mesoporous silica nanoparticles (MEMSN); enhanced permeability and retention (EPR); alternating magnetic fields (AFM); European Union (EU); gadolinium chelates (GC); iron oxide particles (IOP); ultrasmall superparamagnetic iron oxide nanocrystals (USPION); micron-sized iron oxide particles (MIOP); hydrodynamic diameter (D_H_); longitudinal water relaxation time (T_1_); transverse water relaxation time (T_2_); longitudinal relaxivity (r_1_); transverse relaxivity (r_2_); gastrointestinal (GI); half-life (t_1/2_); Japan (JP); Australia (Au); zwitterion-coated exceedingly small superparamagnetic iron oxide nanocrystals (ZES-SPION); glucosamine (GlcN); polyacrylic acid (PAA); dimercaptosuccinic acid (DMSA); bovine serum albumin (BSA); mononuclear phagocyte system (MPS); transferrin receptors (TfR); matrix metalloproteinase (MMP-12); field free region (FFR); mesenchymal stem cells (MSC); magnetic activated cell sorting (MACS); deoxyribonucleic acid (DNA); catalytic antimicrobial robots (CAR); enzyme-linked immunosorbent assay (ELISA); quantitative polymerase chain reaction (qPCR).
